# Neurological complications following pediatric allogeneic hematopoietic stem cell transplantation: Risk factors and outcome

**DOI:** 10.3389/fped.2022.1064038

**Published:** 2022-12-02

**Authors:** Irina Zaidman, Tamar Shaziri, Dina Averbuch, Ehud Even-Or, Yael Dinur-Schejter, Adeeb NaserEddin, Rebecca Brooks, Bella Shadur, Aharon Gefen, Polina Stepensky

**Affiliations:** ^1^ Faculty of Medicine, Hebrew University of Jerusalem, Jerusalem, Israel; ^2^ Department of Bone Marrow Transplantation and Cancer Immunotherapy, Hadassah-Hebrew University Medical Center, Jerusalem, Israel; ^3^ Department of Pediatrics, Hadassah—Hebrew University Medical Center, Jerusalem, Israel; ^4^ Pediatric Intensive Care Unit, Hadassah—Hebrew University Medical Center, Jerusalem, Israel; ^5^ Immunology Division, Garvan Institute of Medical Research, Sydney, NSW, Australia; ^6^ St Vincent’s Clinical School, University of New South Wales, Sydney, NSW, Australia; ^7^ Division of Pediatric Hematology Oncology and Bone Marrow Transplantation, Ruth Rappaport Children’s Hospital, Rambam Medical Center, Haifa, Israel; ^8^ Faculty of Medicine, Technion—Israel Institute of Technology, Haifa, Israel

**Keywords:** neurological complications, hematopoietic stem cell transplantation, survival, neurological sequelae, abnormal imaging, pediatric malignant and nonmalignant diseases

## Abstract

**Background:**

Allogeneic hematopoietic stem cell transplantation (HSCT) is an efficient treatment for numerous malignant and nonmalignant conditions affecting children. This procedure can result in infectious and noninfectious neurological complications (NCs).

**Objective:**

The objective of the study is to examine the incidence, risk factors, and outcomes of NCs in pediatric patients following allogeneic HSCT.

**Methods:**

We performed a retrospective study of 746 children who underwent 943 allogeneic HSCTs in two large pediatric hospitals in Israel from January 2000 to December 2019.

**Results:**

Of the pediatric patients 107 (14.3%) experienced 150 NCs. The median follow-up was 55 months. Noninfectious NCs were more common than infectious NCs (81.3% vs. 18.7%). Factors significantly associated with type of NC (infectious vs. noninfectious) were underlying disease (immunodeficiency vs. malignant and metabolic/hematologic disease) (*p*-value = 0.000), and use of immunosuppressive agent, either Campath or ATG (*p*-value = 0.041). Factors with a significant impact on developing neurological sequelae post-NC were number of HSCT >1 (*p*-value = 0.028), the use of alemtuzumab as an immunosuppressive agent (*p*-value = 0.003), and infectious type of NC (*p*-value = 0.046). The overall survival rate of whole NC-cohort was 44%; one-third of all mortality cases were attributed to the NC. The strongest prognostic factors associated with mortality were older age at HSCT (*p*-value = 0.000), the use of alemtuzumab as an immunosuppressive agent (*p*-value = 0.004), and the existence of neurological sequelae (*p*-value = 0.000). Abnormal central nervous system imaging (*p*-value = 0.013), the use of alemtuzumab as an immunosuppressive agent (*p*-value = 0.019), and neurological sequelae (*p*-value = 0.000) had statistically significant effects on neurological cause of death.

**Conclusion:**

Infectious and noninfectious NCs are a significant cause of morbidity and mortality following allogeneic HSCT in children. Further research is required to better understand the risk factors for different NCs and their outcomes regarding sequelae and survival.

## Introduction

Hematopoietic stem cell transplantation (HSCT) is an efficient treatment for numerous pediatric malignant and nonmalignant disorders. In preparation for the transplant, patients receive high-dose chemotherapy with or without irradiation (conditioning regimen), to destroy diseased cells and to reduce immunologic resistance. Following allogeneic HSCT, patients receive immunosuppressive medications to prevent graft-vs.-host disease (GVHD) (1).

The intensive conditioning, transplantation itself, and immunosuppressive therapy may lead to severe complications in many organs, including the nervous system, and cause neurological complications (NCs)—seizures, stroke, meningitis, and encephalitis—associated with morbidity and mortality (2).

The reported incidence of post-HSCT NCs ranges from 6.5% to 65% (3–12) and varies widely between different centers due to the heterogeneity of study groups and the criteria of NCs (5). NCs can occur secondary to a range of infectious and noninfectious causes, including medication-induced, metabolic, vascular, immune-mediated, or disease-related neurotoxicity (including relapse) (5, 13). Prevalence of infectious NCs is less than that of noninfectious NCs (1, 3, 7, 8, 10, 14). Many factors affect the incidence and severity of NC after HSCT including allogeneic transplantation (1, 4, 5, 9, 11), mismatched donor (3, 4, 9, 12), radiation-based conditioning regimen (1, 8), and GVHD (3–5, 9, 12).

NCs after HSCT can be divided into three groups according to onset (15): early complications during the first month after HSCT, such as seizures and posterior reversible encephalopathy syndrome (PRES) as a consequence of neurotoxic conditioning agents, or infections and bleeding secondary to conditioning-induced pancytopenia (16); intermediate-onset complications, between 1 and 6 months after HSCT, with central nervous system (CNS) infections; and late complications, >6 months after HSCT, like CNS relapse, late chemo-radiotherapy related neurotoxicity, and neurological manifestations of GVHD (1).

The survival rates of patients who develop NCs, both children and adults, are reduced (8, 9, 12, 17); mortality could be attributed to the NCs in more than half of the cases (8, 11); and 27%–43% (5, 7, 18) suffer from long-term neurological sequelae. NCs are often accompanied by neuroimaging changes such as brain edema, intracranial hemorrhage, ring-enhancing lesions or abscess, and cortical dysplasia (19–21).

Our goal was to study a large cohort of pediatric patients from two large pediatric medical centers who underwent allogeneic HSCT and developed NCs, over a period of 20 years. We aimed to examine the rate and risk factors of infectious and noninfectious NCs and their impact on outcomes such as survival and long-term neurological sequelae.

## Materials and methods

### Study population

We performed a retrospective study in two tertiary pediatric hospitals, analyzing the incidence, risk factors, and outcomes of pediatric patients who developed NCs following allogeneic HSCT, between January 2000 and December 2019. All patients younger than 21 years at the time of HSCT, who experienced at least one neurological event during follow-up, were included. We excluded from this study pediatric patients that underwent autologous HSCTs and patients with brain tumors.

The study was approved by the Institutional Review Boards of both centers.

### Data collection

Data were collected from medical records and entered directly into password-protected Microsoft Excel spreadsheets (Redmond, WA, United States). Data included gender, age at HSCT, diagnosis, number of HCSTs, type of donor, conditioning regimen and immunosuppression, type of GVHD, type and timing of NC, investigations of infectious and noninfectious NCs [laboratory, imaging (computed tomography, CT, and magnetic resonance imaging, MRI), and electroencephalography], and neurotoxic potential of medications. To determine if neurological sequelae are present, patients were followed from the time of their initial NC, date of last follow-up, overall survival (OS), and date and cause of death (neurological vs. non-neurological). Patients were evaluated by senior physicians specialized in pediatric HSCTs and pediatric neurologist. Cause of death was determined by senior physicians after reviewing the medical records. Clinical and neurological evaluation at last follow-up of living patients were conducted in HSCT late effects clinics.

### Definitions

We defined three groups underlying diseases: malignant diseases [acute lymphoblastic leukemia (ALL), myelodysplastic syndrome (MDS)/acute myeloid leukemia (AML), chronic myeloid leukemia (CML), juvenile myeloid leukemia (JMML), and Hodgkin and non-Hodgkin lymphomas]; nonmalignant-immunodeficiency [severe combined immunodeficiency (SCID), leukocyte adhesion deficiency (LAD), Wiskott–Aldrich syndrome, Kostmann syndrome, hemophagocytic lymphohistiocytosis (HLH), X-linked lymphoproliferative disease (XLP), Griscelli syndrome, and other rare primary immunodeficiencies]; nonmalignant-other [metabolic: metachromatic leukodystrophy (MLD), adrenoleukodystrophy (ALD), osteopetrosis, and hematological diseases: bone marrow failures: severe aplastic anemia (SAA), pure red cell aplasia, dyskeratosis congenita, Fanconi anemia, and hemoglobinopathies—sickle cell anemia, thalassemia major].

Conditioning regimens were divided into four groups: myeloablative with radiation (total body irradiation with etoposide or cyclophosphamide), myeloablative without radiation (busulfan-based regimen with cyclophosphamide, or melphalan), myeloablative reduced toxicity (treosulfan-based protocol with fludarabine and thiotepa), and reduced-intensity conditioning (RIC) (busulfan/fludarabine; treosulfan/fludarabine).

Serotherapy usage in conditioning regimen was classified as none, anti-thymocyte globulin (ATG), and alemtuzumab.

Donor types were divided into three groups based on HLA-compatibility: matched (10/10), mismatched (8–9/10), and haploidentical (related donors <8/10). Both matched and mismatched groups included related and unrelated donors.

Acute and chronic GVHD (AGVHD and CGVHD, respectively) were graded according to the modified Glucksberg criteria (22) and the revised Seattle criteria (23), respectively.

The etiology of the NCs was determined by signs and symptoms, medications, and microbiological and radiological characteristics. NCs were defined as “infectious” if a pathogen was identified by culture, serology, or polymerase chain reaction (PCR) in tissue of brain or cerebrospinal fluid (CSF) and as “noninfectious” if the patient displayed no clinical symptoms of infection, no organism was identified, and another cause was found.

Neurological sequelae were defined as residual neurological symptoms or damage after the NC, such as persistent convulsive disorder, paresis and plegia, aphasia, brain or optic nerve atrophy, cognitive deterioration, and coma.

### Statistical analysis

To test the association between two categorical variables, *χ*^2^ test and the Fisher's exact test were used. The comparison of a quantitative variable between two independent groups was carried out by the two-sample *t*-test or the nonparametric Mann–Whitney test. The nonparametric test was used for quantitative variables that were not normally distributed. The logistic regression multivariate model was applied to simultaneously assess the effect of several variables on a dichotomous dependent variable. The Kaplan–Meier model with the log-rank test for the comparison of survival curves was used for testing the effect of categorical variables on survival. The Cox regression model was used to test the effect of quantitative variables on survival. This model was also used as the multivariate model that simultaneously assesses the effect of several variables on survival. Both the Cox regression multivariate model and the logistic regression model were carried out using the stepwise, forward, likelihood ratio method. All tests applied were two-tailed, and a *p*-value of 0.05 or less was considered statistically significant.

Statistical analysis was conducted by SPSS software (Chicago, IL, United States).

## Results

### Patient characteristics and incidence of NCs

From January 2000 to December 2019, 746 pediatric patients underwent 943 allogeneic HSCTs in two tertiary pediatric hospitals in Israel. Detailed characteristics of study population are shown in Table 1. One hundred and seven of these patients experienced 150 neurological events, an incidence of 14.34%. Of 107 patients, 57 were transplanted for malignant and 50 for nonmalignant diseases. Median ages at transplant were 7.8 and 9.96 years in infectious and noninfectious group, respectively. Median times from HSCT to NC were 74.5 and 70.5 days in infectious and noninfectious group, respectively (Table 1). Median follow-up period after at least one neurological event was 55 months (range 0.2–199).

**Table 1:** Characteristics of study population.

Characteristics	Total NCs (*n* = 150)	Infectious NCs (*n* = 28, 18.7%)	Noninfectious NCs (*n* = 122, 81.3%)
Sex			
Male	100 (66.7%)	19 (67.9%)	81 (66.4%)
Female	50 (33.3%)	9 (32.1%)	41 (33.6%)
Age at HSCT, median (range), years	9.7 (0.23–21.01)	7.8 (0.23–16.16)	9.96 (0.26–21.01)
Age at neurological complication, median (range), years	10.2 (0.36–21.18)	8.4 (0.44–21.2)	10.4 (0.36–21)
Primary disease			
Malignant	75 (50%)	8 (28.6%)	67 (54.9%)
ALL	35 (23.3%)	3 (10.7%)	32 (26.2%)
MDS/AML	24 (16%)	3 (10.7%)	21 (17.2%)
CML	3 (2%)	2 (7.1%)	1 (0.8%)
JMML	2 (1.3%)	0	2 (1.6%)
Lymphoma	11 (7.33%)	0	11 (9%)
Nonmalignant	75 (50%)	20 (71.4%)	55 (45.1%)
Metabolic	20 (13.3%)	1 (3.6%)	19 (15.6%)
Hematologic	35 (23.3%)	7 (25%)	28 (23%)
Immunodeficiency	20 (13.3%)	12 (42.9%)	8 (6.6%)
Number of HSCT			
1	125 (83.3%)	23 (82.1%)	102 (83.6%)
2	19 (12.7%)	3 (10.7%)	16 (13.1%)
3	6 (4%)	2 (7.1%)	4 (3.3%)
Type of donor			
Matched	79 (52.7%)	13 (46.4%)	66 (54.1%)
Mismatched	43 (28.7%)	11 (39.3%)	32 (26.2%)
Haploidentical	28 (18.7%)	4 (14.3%)	24 (19.7%)
Conditioning regimen			
Myeloablative with radiation	43 (28.7%)	6 (21.4%)	37 (30.3%)
Myeloablative W/O radiation	68 (45.3%)	7 (25%)	61 (50%)
Myeloablative reduced toxicity	13 (8.7%)	7 (25%)	6 (4.9%)
Reduced-intensity conditioning	26 (17.3)	8 (28.6%)	18 (14.8%)
Immunosuppressive therapy			
None	46 (30.7%)	4 (14.3%)	42 (34.4%)
ATG	83 (55.3%)	18 (64.3%)	65 (53.3%)
Alemtuzumab	21 (14%)	6 (21.4%)	15 (12.3%)
AGVHD			
None	66 (44%)	16 (57.1%)	50 (41%)
Stage 1–2	47 (31.3%)	7 (25%)	40 (32.8%)
Stage 3–4	37 (24.7%)	5 (17.9%)	32 (26.2%)
CGVHD			
None	109 (72.7%)	23 (82.1%)	86 (70.5%)
Limited	22 (14.7%)	4 (14.3%)	18 (14.8%)
Extensive	16 (10.7%)	1 (3.6%)	15 (12.3%)
Unknown	3 (2%)	0	3 (2.4%)
Time from HSCT to neurological complication, median (range), days	71.5 (0–2,992)	74.5 (0–1,876)	70.5 (0–2,292)
Abnormal CNS imaging			
Yes	100 (66.7%)	25 (89.3%)	75 (61.5%)
No	50 (33.3%)	3 (10.7%)	47 (38.5%)
Neurological sequelae			
Yes	57 (38%)	17 (60.7%)	40 (32.8%)
No	93 (62%)	11 (39.3%)	82 (67.2%)

ALL, acute lymphoblastic leukemia; MDS, myelodysplastic syndrome; AML, acute myeloid leukemia; CML, chronic myeloid leukemia; CNS, central nervous system; JMML, juvenile myelomonocytic leukemia; ATG, anti-thymocyte globulin; W/O, without; HSCT, hematopoietic stem cell transplant; NC, neurological complications; AGVHD, acute graft-vs.-host disease; CGVHD; chronic graft-vs.-host disease.

### Etiology of NCs

Most NCs were noninfectious, 81.3% (122 out of 150), vs. infectious, 18.7% (28 out of 150) (Table 1). In the noninfectious group, the common causes of NCs were PRES, convulsions, and encephalopathy secondary to multiorgan failure (33.6%, 15.6%, and 15.6%, respectively), followed by CNS relapse and hemorrhage (11.5% and 9.8%) (Table 2). In the infectious group, most common causes for neurological complications were fungal/protozoal infections (mostly toxoplasmosis; 39.3%), followed by viral (35.7%) and bacterial infections (25%) (Table 3).

**Table 2:** Causes of noninfectious neurological complications (122 events).

Cause	Number of events (%)
PRES	41 (33.6)
Convulsions (of unknown cause)	19 (15.6)
Encephalopathy secondary to MOF	19 (15.6)
CNS relapse/deterioration of CNS disease	14 (11.5)
Intracranial hemorrhage	12 (9.8)
Medication/irradiation side effects	5 (4)
Convulsions (metabolic cause)	4 (3.3)
Thrombotic microangiopathy	3 (2.5)
GVHD of brain (suspected)	3 (2.5)
PTLD	2 (1.6)

PRES, posterior reversible encephalopathy syndrome; MOF, multiorgan failure; CNS, central nervous system; GVHD, Graft-vs.-host disease; PTLD, posttransplant lymphoproliferative disorder.

**Table 3:** Causes of infectious neurological complications (*n* = 28).

Cause	Number of events (%)
Bacterial	7 (25)
*Streptococcus pneumoniae*	4 (14.3)
*Mycobacterium*	3 (10.7)
Viral	10 (35.7)
CMV	4 (14.3)
EBV	4 (14.3)
West Nile virus	1 (3.5)
Enterovirus	1 (3.5)
Protozoa/fungi	11 (39.3)
Toxoplasma	8 (28.6)
Candida	1 (3.5)
Aspergillus	1 (3.5)
Cryptococcus	1 (3.5)

CMV, cytomegalovirus; EBV, Epstein–Barr virus.

### Risk factors for developing noninfectious vs. infectious NCs

The underlying disease was significantly correlated (*p*-value = 0.000) with the type of NC: while patients with malignant diseases and patients with metabolic/hematological diseases had more noninfectious NCs (89.3% and 85.5%, respectively), children with immunodeficiencies experienced mostly infectious NCs (60%).

The type of NC was also associated with the conditioning regimen used: noninfectious NCs were significantly more frequent in patients treated by myeloablative protocol, with or without radiation (86% and 89.6%, respectively), compared with those in patients who received either reduced toxicity or intensity regimens (46.2% and 69.2%, respectively; *p*-value = 0.002).

The immunosuppressive agent was another factor associated with the type of NC. When no immunosuppressive was used, patients had the lowest percentage (8.7%) of infectious NCs, compared with patients treated with alemtuzumab or ATG (30% and 21.7%, respectively). Although the significance of this difference was borderline by univariate analysis (*p*-value = 0.069), it reached a significant level when entered into the multivariate model.

We found no correlation between the type of NC (infectious/noninfectious) and sex, age at HSCT, number of HSCT, type of donor, AGVHD or CGVHD, and age at NC and time from HSCT to NC.

Multivariate analysis deemed only immunosuppressive agent (*p*-value = 0.041) and underlying disease (*p*-value = 0.000) as significantly correlated with the type of NC; children treated without immunosuppressive agent (compared with those treated with alemtuzumab) were 7.624 times more prone to develop a noninfectious NC (*p*-value=0.011). Children with metabolic/hematological diseases, and children with malignant diseases, were 14.102 and 16.711 times more likely to develop a noninfectious NC (*p*-value=0.000) (Table 4).

**Table 4:** Multivariate analysis of significant variables associated with neurological complications.

	*P*-Value	OR (95% CI for OR)
Risk factors for developing a noninfectious NC		
Underlying disease	0.000	
Nonmalignant (excluding immunodeficiency)^a^	0.000	14.102 (3.916–50.785)
Malignant^a^	0.000	16.711 (4.699–59.439)
Immunosuppressive agent	0.041	
None^b^	0.011	7.624 (1.580–36.797)
Risk factors for developing neurological sequela		
Number of HSCT	0.028	
Second transplant^c^	0.007	5.290 (1.562–17.919)
Immunosuppressive agent	0.013	
Alemtuzumab^d^	0.003	6.515 (1.881–22.567)
Type of neurological complication-infectious	0.046	2.585 (1.019–6.559)
Risk factors for a neurological cause of death		
Abnormal imaging	0.013	19.380 (1.856–202.370)
Immunosuppressive agent	0.057	
Alemtuzumab^e^	0.019	7.768 (1.395–43.260)
Neurological sequela	0.000	24.162 (4.222–138.282)

	*P*-Value	HR (95% CI for HR)

Survival analysis		
Older age at HSCT	0.000	1.082 (1.034–1.131)
Neurological sequela	0.000	2.693 (1.708–4.245)
Immunosuppressive agent	0.009	
Alemtuzumab^d^	0.004	2.696 (1.377–5.281)

HR, hazard ratio; HSCT, hematopoietic stem cell transplant; NC, neurological complication; OR, odds ratio.

^a^ Compared with nonmalignant, immunodeficiency.

^b^ Compared with alemtuzumab.

^c^ Compared with first transplant.

^d^ Compared with no immunosuppressive agent.

^e^ Compared with ATG.

### Neurological sequelae

Neurological sequelae were developed in 36.4% of children with NCs (Table 5).

**Table 5:** Neurological sequelae.

	Number of children (%)
Neurological complications	107
Neurological sequelae	39 (36.4%)
Coma	15
Persistent convulsive disorder	14
Hemiparesis/paraplegia/quadriplegia	5
Optic nerve atrophy/brain atrophy	3
Aphasia	1
Cognitive deterioration	1

The underlying disease had a statistically significant effect on developing neurological sequelae (*p*-value = 0.019). The highest incidence (65%) was seen in patients in the nonmalignant-immunodeficiency group, to compare with 38.2% and 30.7% in the malignant and nonmalignant-other groups, respectively.

An association was seen between the number of HSCTs and neurological sequelae (*p*-value = 0.028). The highest percentile (63.2%) of sequelae was seen in patients that underwent two transplants, and the lowest percentile (33.6%) of sequelae was seen in patients that underwent one transplant. Half of patients that underwent a third transplant developed neurological sequelae, but the numbers were too small for statistical analysis.

We also found a correlation between the immunosuppressive agent used and neurological sequelae (*p*-value = 0.011). The highest percentile of neurological sequelae (65%) was seen in patients treated with alemtuzumab, compared with 37.3% among those treated with ATG, and 26.1% in patients not receiving any serotherapy.

The type of NC had a significant impact on whether they developed neurological sequelae: 60% of patients with infectious NCs suffered neurological sequelae compared with 32.8% of patients with noninfectious NCs (*p*-value = 0.006).

A correlation was seen between abnormal CNS imaging and sequelae (*p*-value = 0.021). In patients with abnormalities seen in both brain CT and MRI, the incidence of neurological sequelae was 43.2%, vs. 20% in patients with abnormalities only in MRI.

We found no association between developing a neurological sequelae and sex, conditioning regimen, type of donor, CGVHD, age at HSCT, and time from HSCT to NC.

Multivariate analysis of variables associated with neurological sequelae demonstrated that only a number of HSCTs more than 1 [odds ratio (OR) = 5.29, *p*-value = 0.007], alemtuzumab as serotherapy (OR = 6.515, *p*-value = 0.003), and infectious NC (OR = 2.58, *p*-value = 0.046), remained statistically significant (Table 4).

### Survival

The OS of our cohort was 44%. Among patients who developed NCs during or after HSCT, 47 survived with median follow-up period 55 months (range 0.2–199).

Out of the 60 patients who did not survive, 20 died due to neurological causes and 40 died due to other reasons. The mortality rate from neurological reason in our study was 33% of all mortality cases of patients diagnosed with NCs.

There was an association seen between age of patients at HSCT and/or at the NC and survival. The survival period was shorter as the patient was older at the transplant (*p*-value = 0.003) and at the NC (*p*-value = 0.004). The death rate increased by a factor of 1.066 for every year older at the age of HSCT.

**Figure 1:**
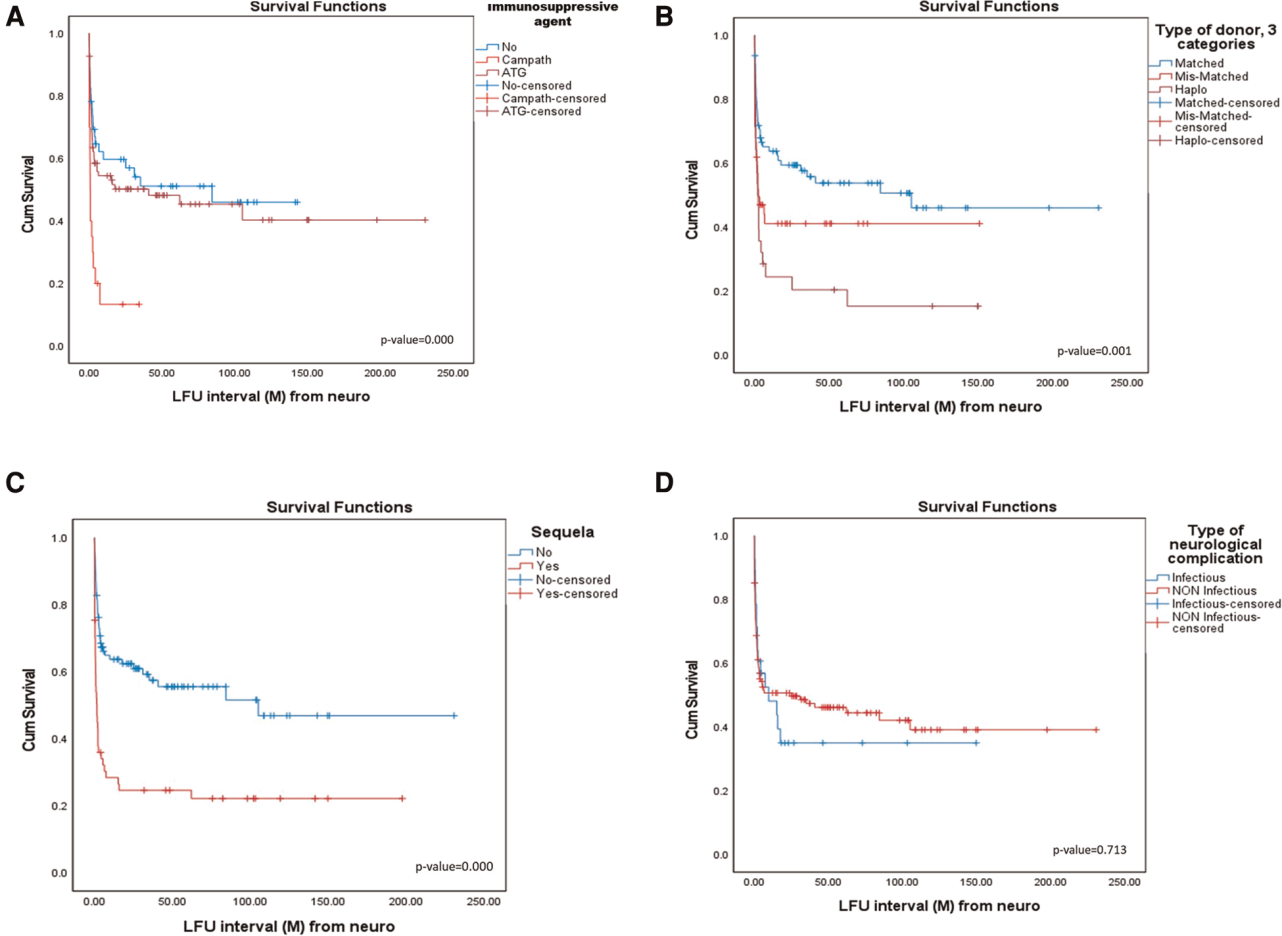
(**A**) Survival according to immunosuppressive agent. (**B**) Survival according to type of donor. (**C**) Survival according to neurological sequelae. (**D**) Survival according to type of neurological complication.

The immunosuppressive agent used had a significant effect on patient's survival (*p*-value = 0.000). The shortest survival was seen in patients treated with alemtuzumab—median 0.77 months, vs. a median of 84.37 months when no immunosuppressive agent was used and 40.87 months in patients treated with ATG (Figure 1A).

The underlying disease had a significant effect on patient's survival (*p*-value = 0.037). The shortest survival was seen in the malignant and nonmalignant-immunodeficiency groups, median 4.4 months, vs. nonmalignant-metabolic and hematologic patients, 84.37 months.

Type of donor had a significant effect on patients' survival (*p*-value = 0.001). The shortest median survival seen in the haploidentical group was 1.87 months, and in the mismatched group was 2.3 months, compared with 105.1 months after matched-donor HSCTs (Figure 1B).

Development of neurological sequelae after NC had a significant impact on patient survival (*p*-value = 0.000). Median survival for patients who developed sequelae was shorter at 1.8 months, compared with those without sequelae at 84.3 months (Figure 1C).

Survival was not correlated with abnormal imaging, conditioning regimen, time from HSCT to neurological complication, type of NC (Figure 1D), sex, underlying disease, AGVHD, and CGVHD.

A multivariable analysis that included all correlated variables demonstrated that age at NC (HR = 1.082, *p*-value = 0.000), neurological sequelae (HR = 2.693, *p*-value = 0.000), and the use of alemtuzumab as an immunosuppressive agent (HR = 2.696, *p*-value = 0.004) had the strongest impact on survival (Table 4).

### Cause of death

An association was seen between number of HSCTs and the cause of death (*p*-value = 0.048). The highest percentiles (100% and 50%) of a neurological cause of death were seen in patients that underwent three or two transplants, respectively, vs. 27.1% in patients after one transplant.

Another association was seen between abnormal imaging and cause of death (*p*-value = 0.000): 47.3% of children with abnormal imaging had a neurological cause of death, compared with 3.7% in patients with normal imaging.

Developing neurological sequelae had a statistically significant impact on the cause of death (*p*-value = 0.000); 64.3% of a neurological cause of death was seen in patients who developed sequelae, vs. 6.3% in patients who did not.

Type of NC was significantly correlated with the cause of death (*p*-value 0.011), with 58.8% of the infectious group having a neurological cause of death vs. 26.2% of the noninfectious group.

The immunosuppressive agent used had a significant effect on patient's cause of death (*p*-value = 0.003). The highest incidence (64.7%) of a neurological cause of death was seen in patients treated with alemtuzumab, compared with patients treated without immunosuppressant or with ATG (33.3% and 18.6%, respectively).

Type of donor was also associated with cause of death. The highest incidence (47.8%) of a neurological cause of death was seen in the haploidentical donor group, compared with matched and mismatched donors (20.0% and 37.5%, respectively).

A correlation was seen between the interval from the patient's first NC to death, and the mortality cause (*p*-value = 0.002), with shorter interval in patients that died due to neurological reason: in the neurological cause of death group, the median NC to death interval was 16 days, while for the non-neurological cause of death group, this interval was 59 days.

We found no correlation between cause of death and sex, underlying disease, conditioning regimen, number of HSCT, age at HSCT, age at neurological complication, and time from HSCT to neurological complication.

Multivariate analysis demonstrated that out of all the statistically significant variables, only abnormal imaging (OR = 19.38, *p*-value = 0.013), sequelae (OR = 24.162, *p*-value = 0.000), and the use of alemtuzumab as an immunosuppressive agent (OR 7.768, *p*-value = 0.019) were statistically significant as risk factors for neurological cause of death (Table 4).

## Discussion

NCs in children who underwent allogeneic HSCT are an important cause of mortality and morbidity, including long-term neurological sequelae. These complications are due to various etiologies: infections (bacterial, fungal/protozoal, or viral encephalitis) or noninfectious (e.g., medication-induced neurotoxicity, metabolic, vascular, immune-mediated, or related to the underlying disease including relapse malignancy) (5, 13). However, research regarding prognostic factors for infectious and noninfectious NCs and sequelae is scarce, especially in children.

The aim of our study was to identify the characteristics, risk factors, and outcomes (neurological sequelae and survival) of pediatric patients who developed infectious and noninfectious NCs during and after allogeneic HSCT.

In our study, we analyzed a large cohort of pediatric patients consisting of 943 allogeneic HSCTs from two major pediatric centers, between the years 2000 and 2019. The incidence of NCs in our cohort was 14.34%. The reported incidence of NCs among children after HSCT varies greatly and ranges from 6.5% to 65% (3–12), which could be attributed to patients' heterogeneity and different definitions of NCs. The incidence of abnormal findings in autopsies is up to 90% (24). A recent Chinese study assessing 196 children (8) found the incidence to be 17%, comparable with our results (and similar to the 14.8% incidence in a recent study on 888 adult allo-transplanted patients) (17). The incidence of NCs after HSCT is higher than in children treated with chemotherapy only (10).

Noninfectious NCs were more than four times frequent than infectious NCs in our cohort. The reported incidence of CNS infections after allogeneic HSCT ranges from 7.5% to 27%, a smaller part from the overall incidence of NCs as described previously (1, 3, 7, 8, 10, 14); our findings are within that range.

Maffini et al. (25) reviewed the timing of NCs in adults after allogeneic HSCT. Early posttransplantation complications were noninfectious, associated mainly with calcineurin inhibitors toxicity (around 50% of all early noninfectious NCs) (1, 5) or with the conditioning regimen, whereas later complications were infectious and associated with immunosuppression status. In our pediatric cohort, the onset of infectious or noninfectious NCs was not statistically different (median 77 and 70 days, respectively). Moreover, in the literature, most NCs tend to occur earlier than 3 months post-HSCT (5, 7–9, 11, 12).

Risk factors for posttransplant pediatric NCs were described, and some of them have conflicting results. Most studies analyzed allogeneic HSCTs, but those that included also autologous transplants found a significant higher incidence in the allogeneic setting (1, 9, 11). Regarding pretransplant factors, underlying malignant diseases, especially leukemia or lymphoma (8, 12) or diseases already involving the CNS (8), radiation-based conditioning (1, 8, 20), and mismatched donor (9, 12) (in earlier works, but not in the recent ones, probably due to better matching processes), were associated with higher risk. GVHD was found to be correlated with NCs (9, 12); a recent retrospective analysis revealed statistically significant correlations between noninfectious NCs and GVHD, specifically between PRES or seizures, and acute GVHD, as well as seizures or stroke, and chronic GVHD; but causal relationships could not be established (4). The above-mentioned large adult study found increased age as the only significant risk factor (17).

In our work, infectious NCs were more frequent in patients who received myeloablative reduced toxicity (53.8%) or RIC (42.3%) regimens, vs. those who were treated with myeloablative protocols, with or without radiation (16.3% and 17.9%, respectively). A possible explanation is that the goal of reduced toxicity/intensity conditioning is tumor eradication and/or destruction of host hematopoiesis *via* immune-mediated effects (using serotherapy such as alemtuzumab or ATG), rather than cytotoxic effect (26). RIC protocols are thus related to higher prevalence of infectious complications.

As expected, infectious type of complications was more frequent in patients who received immunosuppressive agent, either alemtuzumab or ATG. Moreover, some negative outcomes were more frequent in those treated with alemtuzumab: prevalence of neurological sequelae and mortality, as well as neurological cause of death. Alemtuzumab is a monoclonal antibody against the CD52 antigen, expressed in both B and T lymphocytes (27); ATG, however, affects only T lymphocytes (28); immune recovery is slower after treatment with alemtuzumab rather than ATG (29). Avivi et al. (30) showed that adult patients treated with alemtuzumab-based RIC may have a lower risk of developing regimen-related/noninfectious NCs and are more susceptible to infectious NC. We did not find pediatric studies that described the association between the type of conditioning regimen and usage alemtuzumab/ATG and higher incidence of infectious NCs.

We observed the highest incidence (60%) of infectious NCs in the nonmalignant-immunodeficiency group. One reason could be the pretransplant poor immunological status of these patients that increases their susceptibility to infections. Another possible explanation is the prevalent use of RIC regimens used in these patients.

On the other hand, in our study, myeloablative conditioning protocols, with or without radiation, were risk factors for noninfectious NCs. Usually, myeloablative protocols put less emphasis on immunosuppressive effects; however, myeloablative medications such as busulfan and thiotepa, and the exposure to radiation, are known to be toxic to the brain tissue and cerebral vasculature. The incidence of neurotoxicity after busulfan-based conditioning is up to 7% in children (31), and includes seizures, PRES, altered consciousness, and headaches (32). Thiotepa is reported to cause chronic encephalopathy with progressive decline in cognitive and behavioral functions (33). As for radiation, CNS toxicity includes focal cerebral necrosis, neurocognitive deficits, cerebrovascular disease, myelopathy, and secondary neoplasms (34).

Another risk factor for developing noninfectious NCs was the patient's underlying disease being metabolic and hematological. This could be attributed to the neurotoxicity of the busulfan-based myeloablative protocols that are required for most patients with these diseases (31, 32).

Many survivors develop long-term neurological sequelae, including a wide range of neurological deficits, from severe leukoencephalopathy, long-lasting convulsion disorders, to more subtle and mild dysfunction such as learning disorders. Some of these sequelae are accompanied by imaging abnormalities (33). The presence of sequelae reported to be 27%–43% (5, 9, 18). Shin et al. demonstrated the following risk factors to be associated with neurological sequelae: malignancy as the underlying disease, infectious NC occurring after day 100 post-HSCT, and severe MRI abnormalities. Neither radiation nor the intensity of conditioning regimen nor the type of immunosuppressant affected the neurological prognosis (7). We found higher rates of neurological sequelae among patients who suffered an infectious NC rather than noninfectious, 56.8% vs. 31.9%, respectively. This may be explained by the reversible nature of some of the causes of noninfectious NCs post-HSCT, like hydroelectrolytic abnormalities and cyclosporine-associated PRES (35). Like us, Shin et al. (7) demonstrated that in their poor outcome group, there were more instances of malignancy as the underlying disease and infection as the cause of the neurological symptoms. Moreover, treatment with alemtuzumab was found to be a risk factor for developing neurological sequelae, compared with treatment with ATG or without immunosuppressive agent, by the same mechanism of delayed immunological reconstitution as discussed previously. Literature is scarce regarding neurological sequelae following post-HSCT NCs, especially among children; there is no consensus regarding risk factors for sequelae. Kang et al. reported in their pediatric study that 42.5% of neurological episodes had remaining neurological sequelae, 13.7% of them were severe. When they analyzed the sequelae according to etiology, 22.9% of calcineurin inhibitor-related (noninfectious) neurotoxicity had neurological sequelae, vs. 50% of infectious NCs (5).

Developing NCs after a second HSCT was associated with higher rates of neurological sequelae, 5.1 times more likely than after first HSCT. Generally, second transplants are associated with increased toxicity and poorer outcomes than the first HSCT (36, 37), which could be attributed to increased toxicity of a prolonged peritransplant treatment and a more resistant underlying disease. There is not much research regarding NC following second HSCT; however, Shah et al. reported a decreased neurocognitive function after a second HSCT (38). In their study, Mori et al. showed a higher incidence of HHV6-associated encephalitis/myelitis in recipients who underwent two or more HSCTs (39).

In the literature, OS of patients with NCs is inferior to those without NCs (3, 6, 8, 9, 12, 17). OS in our study group was 44%, comparable with current literature that depicts 30%–52.1% rate (3, 5, 6, 8, 9, 11, 17). Uckan et al. (12) found very dismal survival rate of 9% for children presented with *life-threatening* NCs.

In our cohort, the morality due to a neurological cause was 33% of mortality among NCs cases. In the literature, this rate is higher: 50%–61% (8, 11). Kang et al. found that extended chronic GVHD and the presence of neurological sequelae were associated with increased risk of mortality (HR 5.98 and 4.37, respectively) (5). On the other hand, the CNI-associated neurotoxicity was transient, not affecting either the survival outcome (3) or the occurrence of late neurological sequelae (1). We confirmed, in multivariate analysis, that older age at HSCT and the existence of neurological sequelae were significant risk factors for mortality. The death rate increased by a factor of 1.062 for every year older at the age of HSCT. There are conflicting results regarding the effect of age at transplant on mortality in literature. Socié et al. demonstrated lower mortality in patients younger than 15 years at HSCT (40), while Martin et al. did not identify a relationship between age at transplant and late mortality (41). Older age at transplant was also associated with a worse health-related quality of life (42). However, these studies do not discuss the relation between age at HSCT and mortality specifically after NCs. Regarding neurological sequelae, we suppose that developing sequelae indicates a more severe and irreversible CNS damage, increasing the risk of mortality.

Along the timeline of the neurological toxicity, the clinical picture could be accompanied by neuroimaging findings—starting from CT changes during the acute phase (19) to long-term alterations of the MRI (20, 21); the impact of these changes on the patients' outcomes is not fully elucidated. A recent study of Shin et al. (7) found that among 91 transplanted children with NCs, MRI changes were present in 56%. Zając-Spychała et al. described long-term imaging and functional outcomes of pediatric patients with acute lymphoblastic leukemia after radiation-based HSCT, compared with nontransplanted children, and found that the former had significant decline in both brain structures (as demonstrated by MRI) and cognitive performance tests (20). In our study, additional risk factor for a neurological cause of death included abnormal imaging. We assume that the existence of abnormalities seen in imaging is implying a more severe CNS damage, thus resulting in death due to a neurological cause. Regarding the imaging modality, there is a marked difference between abnormal findings seen in CT and in MRI. MRI shows *functional* changes in CNS, such as the mostly-reversible PRES, the main cause for NCs after pediatric allogeneic HSCT. CT however shows *structural* CNS damages (abscesses, cerebrovascular complications, etc.) that are more likely to leave permanent sequelae (43). That correlates with the higher percentile of a neurological cause of death among children with abnormalities seen on CT, in comparison to children with abnormalities seen on MRI.

Our study included symptomatic patients; a future research direction could be screening of all transplanted children for presymptomatic changes, both by imaging and cognitive tests, as described above for leukemic patients (20), and by newer neuro-electrophysiological tools like evoked potentials, as already done by Kroczka et al. (44–46) in leukemic patients (with emphasis on irradiated patients (45). With these modern tools, the group of patients presenting NCs would likely be larger.

Our study has some strengths. First, this is one of the largest and longest studies that analyzed the characteristics of pediatric patients with NCs following allogeneic HSCT, compared infectious vs. noninfectious NCs, and defined risk factors for developing complications, neurological sequelae, and the associated mortality.

In our study, we found and described the correlations between type of conditioning regimen and immunosuppressive agent, and type of NCs (infectious vs. noninfectious) in pediatric patients, which have not been noted before in literature. We also described that late-onset neurological sequelae are associated with alemtuzumab, and with infectious NCs.

We recognize the study's limitations, mainly its retrospective nature and involving only two centers.

In conclusion, infectious and noninfectious NCs are a significant cause of morbidity and mortality following allogeneic HSCT in the pediatric population. Further multicenter prospective research is required for a deeper understanding of the individual risk factors for different NCs and their different outcomes regarding sequelae and mortality. This knowledge could be implemented in clinical practice for developing preventing strategies and early detection.

## Data Availability

The original contributions presented in the study are included in the article/Supplementary Material, further inquiries can be directed to the corresponding author.
